# Quality of life in patients treated with first-line antiretroviral therapy containing nevirapine or efavirenz in Uganda: a prospective non-randomized study

**DOI:** 10.1186/s12913-015-0959-0

**Published:** 2015-07-28

**Authors:** Doris Mutabazi Mwesigire, Albert W. Wu, Faith Martin, Achilles Katamba, Janet Seeley

**Affiliations:** Department of Medicine, Makerere College of Health Sciences, P.O.Box 7072, Kampala, Uganda; Department of Health Policy and Management, John Hopkins Bloomberg School of Public Health, Baltimore, Maryland USA; Department of Psychology, University of Bath, Bath, United Kingdom; MRC/UVRI Uganda Research Unit on AIDS, Uganda Virus Research Institute, Entebbe, Uganda

**Keywords:** Quality of life, HIV/AIDS, Efavirenz, Nevirapine

## Abstract

**Background:**

The goal of antiretroviral therapy (ART) is to suppress viral replication, reduce morbidity and mortality, and improve quality of life (QoL). For resource-limited settings, the World Health Organization recommends a first-line regimen of two-nucleoside reverse-transcriptase inhibitors and one non-nucleoside transcriptase inhibitor (nevirapine (NVP) or efavirenz (EFV)). There are few data comparing the QoL impact of NVP versus EFV. This study assessed the change in QoL and factors associated with QoL among HIV patients receiving ART regimens based on EFV or NVP.

**Methods:**

We enrolled 640 people with HIV eligible for ART who received regimens including either NVP or EFV. QoL was assessed at baseline, three months and six months using Physical Health Summary (PHS) and Mental Health Summary (MHS) scores and the Global Person Generated Index (GPGI). Data were analyzed using generalized estimating equations, with ART regimen as the primary exposure, to identify associations between patient and disease factors and QoL.

**Results:**

QoL increased on ART. The mean QoL scores did not differ significantly for regimens based on NVP versus EFV during follow-up for MHS and GPGI regardless of CD4 stratum and for PHS among patients with a CD4 count >250 cells/μL. The PHS-adjusted β coefficients for ART regimens based on EFV versus NVP by CD4 count strata were as follows: −1.61 (95 % CI −2.74, −0.49) for CD4 count <100 cells/μL; 0.82 (0.22, 1.43) for CD4 count 101–250 cells/μL; and −1.33 (−5.66, 3.00) for CD4 count >250 cells/μL. The corresponding MHS-adjusted β coefficients were as follows: −0.39 (−1.40, 0.62) for CD4 < 100 cells/μL; 0.16 (−0.66, 0.98) for CD4 count 101–250 cells/μL; and −0.75 (−2.01, 0.51) for CD4 count >250 cells/μL. The GPGI-adjusted odds ratios for EFV versus NVP were 0.51 (0.25, 1.04) for CD4 count <100 cells/μL, 0.98 (0.60, 1.58) for CD4 count 101–250 cells/μL, 1.39 (0.66, 2.90) for CD4 > 250 cells/μL. QoL improved among patients on EFV over the 6-month follow-up period (MHS p < 0.001; PHS p = 0.04, p = 0.028). Overall, patients with depression (PHS p < 0.001; GPGI p < 0.001) had lower scores and women had lower MHS (on NVP, p = 0.001). Other factors associated with lower QoL included alcohol use, low education level and advanced HIV disease.

**Conclusions:**

ART improves QoL. The results support use of either NVP or EFV. Patients initiating ART should be assessed for depression and managed appropriately. Women may require extra support to improve their QoL.

## Background

The World Health Organization (WHO) recommendation for combination antiretroviral therapy (ART) for HIV is two-nucleoside reverse-transcriptase inhibitors (zidovudine (AZT)/abacavir plus lamivudine (3TC) or nucleotide tenofovir (TDF) plus 3TC) and a non-nucleoside reverse-transcriptase inhibitor: efavirenz (EFV) or nevirapine (NVP) [1]. Uganda adopted these guidelines, and regimens based on EFV or NVP are used for first-line treatment [2]. Although EFV and NVP have comparable clinical and virological efficacy [3–5], there are several differences between the regimens in terms of other clinical outcomes and patient quality of life (QoL). EFV was reported to produce stronger virological responses than EFV among Ugandan patients receiving a first-line ART regimen [6]. Furthermore, a systematic review reported that an EFV-based first-line regimen was less likely to lead to virological failure than a NVP-based regimen [7].

The majority of clinical studies have focused on the immunological and virological outcomes associated with EFV and NVP. However, with the availability of ART, there is no doubt that survival has been improved. In Uganda, HIV/AIDS patients reportedly now have a life expectancy similar to that of the general population [8]. For this reason, and with the increased availability of different drugs, there is an increasing focus on QoL in selecting a regimen [9]. Studies of QoL associated with different ART regimens have had inconsistent results. One study that assessed QoL among ART- naïve patients receiving AZT and didanosine with or without NVP found better QoL among patients on two drugs. However, there was a better clinical and virological outcome with the triple therapy [10]. In a study among virologically suppressed patients receiving a protease inhibitor that was replaced with EFV or NVP, sustained viral suppression was reported for both drugs and both groups reported significant improvement of QoL [11]. Casado et al. [12] reported no differences at baseline in demographic and clinical variables between patients receiving AZT and 3TC with NVP or nelfinavir. In addition, there were no differences in QoL scores after 12 months of therapy [12]. However, there was a trend towards improved Physical Health Summary (PHS) and Mental Health Summary (MHS) scores for patients on NVP [12]. It was also reported that regimens containing EFV and/or NVP led to a similar increase in QoL [4]. It is critical to understand the QoL outcomes with the current ART regimens used in resource-limited settings.

Other factors have been associated with PHS, including baseline viral load concentration ≥100,000 copies/ml and decline in plasma viral load. An increase in MHS has been associated with a decline in viral load. The presence of an adverse event during follow-up has also been associated with declines in both the MHS and PHS [4]. Adherence to ART has been reported to be associated with QoL. Patients with a record of 100 % adherence to therapy had higher QoL scores after one year of therapy than those with lower adherence (80 %), and those with adherence of less than 80 % had the worst QoL scores [13]. Depression, younger age and low adherence to therapy were reported as predictors of virological failure at baseline among patients receiving regimens based on EFV and NVP [14]. Also, good QoL and depression scores were reported among African patients receiving regimens based on EFV or protease inhibitors [15].

In summary, studies suggest similar virologic and clinical outcomes for regimens based on NVP or EFV, but findings for QoL outcomes have been inconsistent. With the increased access to ART in sub-Saharan Africa, we need to ensure both good clinical outcomes and optimal QoL. Research is needed to improve understanding of the impact on QoL of different treatment options. There is limited research on the differences in QoL between the recommended ART regimens and sparse data on the different factors associated with QoL in relation to ART regimens. The aim of this study was to assess the effect of either NVP or EFV based regimen on QoL the other factors associated with QoL and these ART regimens. Our findings will help to inform strategies to optimize patient outcomes.

## Methods

### Study participants

We enrolled 640 patients from an outpatient HIV clinic at the Ugandan National Referral Hospital between April 2012 and December 2013. Patients were eligible for the study if they were starting ART on the day of the interview, were prescribed either of the following regimens (TDF plus 3TC plus EFV/NVP or AZT plus 3TC plus EFV/NVP), had a CD4 count <350 and were 18 years or older. Owing to the variable nature of CD4 count with time of day, fatigue, stress or infections [16], patients with CD4 counts between 350 and 400 who were ready to commence treatment were given ART. Patients who did not consent, who were pregnant or were too ill were excluded from the study. Owing to the increased risk of hepatotoxicity, female patients with CD4 > 250 cells/μL were started on an EFV regimen and patients with a CD4 count <250 cells/μL and not co-infected with tuberculosis were given a NVP-based regimen since it is cheaper than the EFV-based regimen. Patients co-infected with hepatitis B, regardless of CD4 count, were also initiated on ART. Patients who had had abnormal renal function tests were prescribed AZT instead of TDF.

### Study design

This was a prospective cohort study to assess QoL and factors associated with QoL among patients receiving ART regimens based on NVP or EFV. Patients were followed up for six months and were assessed at baseline, three months and six months.

### Ethical approval

The study was approved by Makerere University School of Medicine, Research and Ethics Committee and the Uganda National Council for Science and Technology. All participants provided written, informed consent prior to participation in the study.

### Measures

Three patient-reported outcome measures were used. First, the Medical Outcomes Study (MOS-HIV) Health Survey was used to collect data on health-related QoL (HRQoL). It is a disease-specific measure that has been widely used [17]. MOS-HIV has been validated in Uganda and is useful in measuring HRQoL in patients with HIV/AIDS [18–20]. Second, the Global Person Generated Index (GPGI) was used to assess global QoL, as perceived by the participant. It has been validated in Ethiopia, Thailand and Bangladesh and found to be reliable in measuring QoL [21]. Third, the Center for Epidemiological Studies Depression scale (CES-D), which has been successfully used in Ugandan settings [22, 23], was used to measure depression. A 3-day self-report was used to measure adherence and a score ≥95 % was reported as adherent and <95 % reported as non-adherent. We used a high cutoff of 95 % for adherent versus non-adherent because higher levels of adherence at >95 % have been proved to achieve higher virological suppression [24,25]. A study among Ugandan HIV patients reported no significant differences in adherence levels while comparing various adherence measures including three-day self-report, visual analog scale, electronic medication monitoring and unannounced pill count [26].

### Independent variables

We collected data on sociodemographic characteristics including sex, age, religion, monthly income, employment status, education level, marital status and social behavioral characteristics (smoking, use of alcohol and social support). Information was documented on clinical characteristics such as WHO HIV stage, opportunistic infection, diagnosis of clinical depression, toxicity that warranted change of regimen, adherence to ART and CD4 + T lymphocyte counts. Blood was drawn at baseline and at the six-month visit for the CD4 cell count for all patients. All patients were screened for depression using CES-D at each of the three visits. We used a cutoff score of at least 16 to suggest probable depression as recommended by the author of CES-D [27].

### Dependent variables

The MOS-HIV subscales were transformed into two summary scores, PHS and MHS [28], normalized to a T-score with a mean of 50 and standard deviation (SD) of 10. GPGI is a subjective measure that enables individuals to rate their own QoL. It is scored on a scale of 0–100. A higher score indicates better global QoL. Population scores for subjective measures such as GPGI range between 60 and 80 [29]. As GPGI was negatively skewed, with a cutoff tail at a score of 60, we categorized a GPGI score of <60 as low and ≥60 as high [29].

### Statistical analysis

To identify determinants of QoL among patients on an EFV-based ART regimen and those receiving a NVP-based regimen, we analyzed the two groups separately. Descriptive statistics and frequencies were determined for all sociodemographic, clinical and outcome variables. For comparisons between the two groups we used chi-square tests for categorical variables, and student’s t-tests, Spearman’s correlation and Wilcoxon’s rank sum tests for continuous variables. We determined effect size as recommended by Cohen [30]. We compared the QoL scores between the two groups over the follow-up period unadjusted and adjusted for potential cofounders, with a p value <0.20 for PHS and GPGI and a p value <0.10 for MHS. The cutoff values were decided after the univariate analysis but before building the multivariate analysis model. We used different cutoffs in order to limit the total number of variables in the model to not more than 10 and hence a stable model [31]. Owing to the differences in CD4 count at baseline among patients given either EFV or NVP, we stratified by baseline CD4 count (<100, 101–250 and >250) to understand better the differences in QoL due to CD4 count and regimen. In each stratum, we also tested for interaction between time and ART regimen. Finally, we also tested for interaction between baseline CD4 count and time in the PHS, MHS and GPGI models.

For the normally distributed outcome variables, we used univariate linear regression with generalized estimating equations (GEE) to identify factors associated with PHS and MHS. All variables determined to be significantly associated with PHS and MHS in univariate models at p < 0.20 and p < 0.10, respectively, were considered in the final multivariate models. Sex and age were considered as forced variables in the final multivariate models. Depression was not included in the MHS model to avoid collinearity since MOS-HIV also covered depression.

We then ran two sets of models: one set with only the forced variables, and the other set with the significant p values (cutoff 0.20 for PHS and 0.10 for MHS). We observed the standard errors for the forced variables and found them to be approximately the same in the two models; these were the final models without multi-collinearity. To further test for collinearity, each of the other variables was dropped from the model and we observed changes in the standard errors for the forced variables. We used logistic regression with GEE to identify demographic, behavioral and clinical characteristics associated with GPGI score at univariate and multivariate analysis. All factors with p < 0.20 at univariate analysis were included in the final multivariate model. The same process was used to build the final multivariate model, as was done for MHS and PHS. The value p < 0.05 was used as a cutoff for the determinants of QoL at multivariable analysis. We tested for interaction between ART regimen and/or time and the significant factors at multivariate analysis. All statistical analyses were conducted using Stata 12.1 (StataCorp 4905 Lakeway Drive college station,Texas, USA).

## Results

Six hundred and forty patients were enrolled in the study, 481 (70 %) were initiated on an EFV-based ART regimen and 159 (30 %) on a NVP-based regimen. Of the total enrolled, 420 (66 %) were women. The mean CD4 change (cells/μL) from baseline to six months was 182 (SD 148.5). The patients on EFV had a higher mean CD4 change (189.8, SD 7.2) than patients on a NVP regimen (160.2, SD 11.5) p = 0.04 (Table 1). There were no significant differences in adherence levels at three months and six months by regimen type. At the three-month visit, 95 % of patients on NVP had an adherence ≥95 % and 93 % of patients on EFV had an adherence score ≥95 % (p = 0.496). By the six-month visit, 94 % of the patients on NVP had an adherence ≥95 % and 93 % of the patients on the EFV regimen had adherence ≥95 % (p = 0.922).

**Table 1:** Baseline sociodemographic and clinical characteristics by ART regimen

Variable	NVP based regimen n (%)	EFV based regimen n (%)	P value
Sex			
Male	65 (41)	155 (32)	0.05
Female	94 (59)	326 (68)	
Age	33.2 (7.68)	33.3 (8.00)	0.88
Education level			
Primary or less	80 (50)	243 (50)	
Secondary	63 (40)	192 (40)	0.98
Apprenticeship	12 (8)	32 (7)	
Tertiary	4 (2)	14 (3)	
Income per month			
(USD)			
<20	49 (31)	146 (30)	0.97
20–60	44 (28)	129 (27)	
>60	66 (41)	206 (43)	
Marital status			
Married	103 (65)	275 (57)	
Separated/divorced	36 (23)	124 (25)	0.30
Single	12 (7)	41 (9)	
Widowed	8 (5)	41 (9)	
Religion			
Christian	124 (78)	397 (83)	0.03
Moslem	28 (18)	79 (16)	
others	7 (4)	5 (1)	
Employment status			
Employed	137 (86)	386 (80)	0.09
Unemployed	22 (14)	95 (20)	
Social support			
Yes, family	126 (79)	405 (84)	0.18
Yes, others	29 (18)	60 (13)	
None	4 (3)	16 (3)	
Alcohol consumption			
Yes	45 (28)	107 (22)	0.12
No	114 (72)	374 (78)	
Smoking			
Yes	6 (4)	18 (4)	0.57
No	153 (96)	96 (96)	
Opportunisticinfection			
Yes	52 (33)	153 (32)	0.83
No	107 (67)	328 (68)	
WHO stage			
1&2	135 (85)	378 (79)	
3&4	24 (15)	103 (21)	0.08
CD4 count (cells/μL)			
<100	48 (30)	68 (14)	1
101–250	93 (59)	96 (20)	0.08
>250	18 (11)	317 (66)	<0.001
			Overall <0.001
CD4 count (cells/μL)	166 (90, 218)	292 (208, 331)	<0.001
Depression			
No depression	103 (65)	310 (64)	
Probable depression	56 (35)	171 (36)	0.94

Among patients who received EFV, 18 (2.8 %) had drug reactions that warranted a change of regimen, 10 (2.1 %) changed from EFV, eight (1.7 %) had hypersensitivity skin reactions, and two (0.4 %) had severe dizziness. Of the eight patients (5 %) who changed from the NVP-based regimen, two (1.3 % of all patients who had received NVP) had Steven Johnson’s syndrome and the rest (3.8 %) had a hypersensitivity rash. Other clinical and sociodemographic characteristics are shown by ART regimen in Table 1.

Regardless of ART regimen, there was a trend towards an increase in the mean QoL scores from baseline to the six-month visit; PHS and MHS were normally distributed and presented as means and GPGI was negatively skewed presented as medians (Table 2). The improvement in QoL while on ART was further confirmed by the effect sizes (change in score/SD; effect size for PHS = 0.8/5.0 = 0.16, MHS = 0.8/4.8 = 0.17) and the correlation for GPGI was 0.35. The effect sizes for PHS and MHS were approximately 0.2, which is referred to as a “small effect” by Cohen, and a correlation of 0.35 is equivalent to “a medium to large effect size” [30]. There were no significant differences between MHS and GPGI scores over six months between NVP and EFV before and after adjusting for potential confounders as summarized in Table 3. However, there was a decrease by 1.6 among patients on the EFV-based regimen with CD4 < 100, and a protective effect of 0.82 among patients with CD4 counts between 101 and 250 on EFV. Similar to MHS and GPGI findings, there was no difference in PHS for patients with CD4 count >250. There was no evidence of interaction between ART regimen and study visit in the PHS model, MHS model and the GPGI model, regardless of the CD4 count strata (Table 3). There was interaction between baseline CD4 count and time in the PHS model (p = 0.003) and borderline evidence with the GPGI model (p = 0.05) but no evidence of interaction in the MHS model (p = 0.28).

**Table 2:** Mean/median QoL scores at baseline, month 3 and month 6 visits

QoL summary score	Baseline visit	Month 3 visit	Month 6 visit	Change from baseline to month 6 (SD/correlation) p value
	Mean (SD) or median (IQR)	Mean (SD) or median (IQR)	Mean (SD) or median (IQR)	
PHS	46.5 (4.6)	47.1 (3.9)	47.3 (3.8)	0.8 (5.0)
				p = 0.0036
MHS	46.4 (4.5)	47.4 (3.5)	47.2 (3.3)	0.8 (4.8)
				p = 0.0002
GPGI	71.7 (55, 85)	71.67 (57, 85)	73.3 (60,87)	r = 0.35
				p < 0.001

**Table 3:** Comparison of QoL scores between regimens based on nevirapine or efavirenz over the follow-up period using GEE

QoL summary score	β coefficient (95 % CI)	β coefficient (95 % CI)
	OR (95 % CI)	OR (95 % CI)
	unadjusted	Adjusted
PHS		
CD4 count <100 cells/μl		
NVP	Ref	Ref
EFV	−1.61(−2.82 to −0.40)	−1.61 (−2.74 to −0.49)^a^
		P = 0.11^d^
CD4 count 101–250 cells/μl		
NVP	Ref	Ref
EFV	0.17 (−0.70 to 1.05)	0.82 (0.22 to 1.43)^a^
		P = 0.76^d^
CD4 count >250 cells/μl		
NVP	Ref	Ref
EFV	0.97 (−0.35 to 2.30)	−1.33 (−5.66 to 3.00)^a^
		P = 0.90^d^
MHS		
CD4 count <100 cells/μl		
NVP	Ref	Ref
EFV	−0.18 (−1.18 to 0.82)	−0.39 (−1.40 to 0.62)^b^
		P = 0.91^d^
CD4 count 101–250 cells/μl		
NVP	Ref	
EFV	−0.09 (−0.93 to 0.75)	0.16 (−0.66 to 0.98)^b^
		P = 0.78^d^
CD4 count >250 cells/μl		
NVP	Ref	Ref
EFV	−0.84 (−2.09 to 0.42)	−0.75 (−2.01 to 0.51)^b^
		P = 0.65^d^
GPGI		
CD4 count <100 cells/μlEFV versus NVP		
	−0.59 (0.31 to 1.13)	0.51 (0.25 to1.04)^c^
		P = 0.54^d^
CD4 count 101–250 cells/μl		
EFV versus NVP	0.85 (0.54 to 1.33)	0.98 (0.60 to 1.56)^c^
		P = 0.08^d^
CD4 count >250 cells/μl		
EFV versus NVP	1.30 (0.66 to 2.56)	1.39 (0.66 to 2.90)^c^
		P = 0.08^d^

^a^adjusted for age, study visit, diagnosis of depression, income per month, WHO stage, education level and opportunistic infection

^b^adjusted for sex, age, study visit, WHO stage, marital status, social support and level of education

^c^adjusted for sex, age, study visit, WHO stage, education level, income per month, depression, employment status, alcohol use, social support and opportunistic infection

^d^P value for interaction term (time*ART regimen)

### Factors associated with PHS among patients receiving a NVP-based ART regimen

Depression was the only factor significantly associated with PHS among patients receiving a NVP-based regimen at both univariate and multivariate analysis (adjusting for study visit, sex, age, diagnosis of depression, income per month, WHO stage, education level, baseline CD4 count and opportunistic infection). People living with HIV/AIDS (PLHA) receiving a NVP-based ART regimen with probable depression (score ≥16) had on average 1.64 lower PHS than those without depression (−1.64, 95 % confidence interval [CI] −2.48 to −0.79; p < 0.001). There was no evidence of interaction between depression and ART regimen (p = 0.89).

### Factors associated with PHS among patients receiving an EFV-based ART regimen

The following factors were associated with PHS among patients receiving an EFV-based ART regimen: study visit, level of education, WHO stage and probable depression. PHS improved from baseline to six months (p = 0.04). In multivariate analysis (adjusting for study visit, sex, age, diagnosis of depression, income per month, WHO stage, education level, baseline CD4 count and opportunistic infection), there was a 0.50 mean increase in PHS at six months compared with baseline visit (95 % CI 0.06 to 0.93). Patents with higher education level had an overall increase in PHS (p = 0.005). For example, patients with tertiary education receiving an EFV-based regimen had a 2.16 mean increase in PHS compared with patients with a primary or no education (95 % CI 0.66 to 3.65). A patient with WHO stage 3&4 compared with those graded as WHO stage 1&2 on average had a 0.86 lower PHS (95 % CI −1.47 to −0.25). Depression was negatively associated with PHS among patients receiving EFV, and PLHA with probable depression had a 1.67 lower mean PHS than patients without depression (95 % CI −2.13 to −1.21). There was no evidence of interaction between WHO stage and ART regimen (p = 0.08), level of education and ART regimen (p = 0.80), study visit and ART regimen (p = 0.90) and depression and ART regimen (p = 0.88). There was evidence of interaction between WHO stage and time (p = 0.0002) and ART regimen and baseline CD4 count (p = 0.003).

### Factors associated with MHS among patients receiving a NVP-based ART regimen

A woman on a NVP-based ART regimen compared with a man had on average a 1.57 point lower MHS score (95 % CI −2.40 to −0.73). Sex was the only factor significantly associated with MHS among patients receiving a NVP-based ART regimen. The factors adjusted for in the multivariate model were sex, age, study visit, WHO stage, marital status, social support, baseline CD4 count and level of education.

### Factors associated with MHS among patients receiving an EFV-based ART regimen

Study visit was the only factor associated with MHS among patients on an EFV-based ART regimen. There was an improvement in MHS (p < 0.001) over time. Patients had a mean increase of 1.04 MHS at the three-month visit compared with the baseline visit (95 % CI 0.62 to 1.46), and a 0.7 point increase in MHS at the six-month visit compared with the baseline visit (95 % CI 0.33 to 1.17). There was no evidence of interaction between the study visit and ART regimen (p = 0.90). The factors adjusted for in the multivariate model were sex, age, study visit, WHO stage, marital status, social support, baseline CD4 count and level of education.

### Factors associated with GPGI among patients receiving a NVP-based ART regimen

For the GPGI score, depression was the only statistically significant predictor in multivariate analysis. A patient on a NVP regimen with probable depression (score ≥16) had 0.31 lower odds of having a high GPGI score (95 % CI 0.19 to 0.51) compared with a patient without depression. There was no evidence of interaction between ART regimen and depression (p = 0.48) and time and depression (p = 0.16). The other variables in the multivariate model included sex, age, study visit, WHO stage, education level, income per month, depression, employment status, alcohol use, social support and baseline CD4 count.

### Factors associated with GPGI among patients receiving an EFV-based ART regimen

Several factors were associated with the GPGI score among patients on an EFV-based regimen: study visit, alcohol consumption and depression. There was a significant overall association of odds of a high GPGI score over time from baseline visit to the six-month visit (p = 0.028). The odds of a high GPGI score at month six compared with baseline were 1.34 (95 % CI 1.01 to 1.77). In addition, in patients taking an EFV-based ART regimen, the odds of a high GPGI were 1.69 (95 % CI 1.18 to 2.42) for those who did not consume alcohol compared with those who did. Patients with probable depression had odds of 0.43 (95 % CI 0.33 to 0.55) for high GPGI score compared with patients without depression. There was no evidence of interaction between ART regimen and alcohol consumption (p = 0.56) and ART regimen and time (p = 0.61). The following variables were included in the multivariate model: sex, age, study visit, WHO stage, education level, income per month, depression, employment status, alcohol use, social support, opportunistic infection and baseline CD4 count.

In summary, depression and sex were associated with QoL scores among patients on the NVP-based regimen and study visit, probable depression, level of education, WHO stage and alcohol use were associated with QoL scores among patients on the EFV-based regimen. Study visit was significant among patients receiving EFV for all the QoL measures, and depression was significant both for PHS and GPGI among patients receiving NVP. Table 4 summarizes all the covariates included in the models.

**Table 4:** Variables included in the multivariate models

PHS model	MHS model	GPGI model
Sex	Sex	Sex
Age	Age	Age
Study visit	Study visit	Study visit
Baseline CD4	Baseline CD4	Baseline CD4
WHO stage	WHO stage	WHO stage
Depression	-	Depression
Income per month	-	Income per month
Level of education	Level of education	Level of education
Opportunistic infection	-	Opportunistic infection
-	Marital status	-
-	Social status	Social status
-	-	Employment status
-	-	Alcohol use

## Discussion

The objective of this study was to determine the factors associated with QoL among HIV patients receiving a first-line ART based on EFV or NVP, and to compare the pattern of results for people on the two medication regimens. There was a statistically significant improvement in QoL scores while on ART, with small to medium/large effect sizes observed. Effect sizes of this magnitude have been interpreted as “a real effect of ART on QoL scores that can be seen through a study to obvious effect” [32].

The differences in QoL scores were small compared with what has been reported in other studies [4, 33]. However, the effect sizes observed (ranging between 0.23 and 0.37) were similar to those found by Cohen [30] and are of a magnitude that has been shown to be related to clinically important differences [34]. Of note, clinically important differences are specific and may differ by study population owing to different patient characteristics such as severity of illness and social economic status. Similarly, some studies in developed countries have also reported an improvement in QoL while on ART [4]. These patients were followed up for a period of 48 weeks. In addition, two longitudinal studies in developing countries have reported an improvement in QoL while on ART [35, 36].

The above studies reported greater differences in QoL than our study, possibly because of the longer time of follow-up. In addition, the majority of participants in these studies were WHO stage 3&4 and likely to have greater changes in QoL, whereas in our study the majority of patients were stage 1&2 and followed up for only six months. These changes would be more comparable to our findings if the effect sizes had been reported. The interaction between baseline CD4 count and time indicates that PHS may change with time while on ART in relation to baseline CD4 count. The borderline significant interaction term between CD4 count and GPGI and lack of evidence for interaction between CD4 count and MHS show that mental health and global QoL may not be dependent on CD4 changes with time. Further to this, there were no significant differences in change in MHS and GPGI QoL scores over time when comparing the EFV and NVP regimens. Patients with CD4 count <100 cells/μL and on EFV had a decrease in PHS. Those with a CD4 count between 101 and 250 cells/μL had an improvement in PHS; however, there was also no difference in PHS between EFV and NVP regimens for patients with CD4 > 250 cells/μL. Generally, there was some improvement in MHS and PHS from baseline to six months among patients on an EFV-based regimen. There was no evidence of interaction between regimen and time. EFV has been reported to improve QoL in other settings [37]. Although there was higher CD4 count recovery in the EFV group than the NVP group, the EFV group had a higher baseline CD4 count, which a known predictor of CD4 recovery [38]. Thus, this change could not be attributed to the use of EFV alone.

There were two severe reactions among patients on the NVP-based regimen (two cases of Steven Johnson’s syndrome). No severe neurological reactions to EFV were reported (although two patients reported severe dizziness). A higher proportion of NVP patients than EFV patients required changing the regimen. Although we did not examine the relationship between adverse events and QoL, results from a qualitative sub study of this population revealed minimal interference with QoL related to side effects [39]. Furthermore, in a large randomized study the side effects due to NVP did not affect the overall QoL [4]. The improvement in QoL and less side effects for patients on the EFV regimen suggests that, where possible, EFV may be preferred to NVP except in patients with a very low CD4 count (<100 cells /μL).

To our knowledge, this study is the first to assess QoL and factors associated with QoL among HIV patients receiving ART regimens based on EFV and NVP in sub-Saharan Africa. The majority of studies have assessed virological outcome and not QoL. For instance, EFV has shown advantages over NVP in some studies, including a lower risk of virological failure [3], greater efficacy [40, 41], better adherence levels [41, 42], greater ease of administration with once a day dosing, and less frequent severe rash and hepatotoxicity [43]. Although similar mortality rates for EFV and NVP were reported in South African patients, those on EFV had higher viral suppression after six months on therapy and were less likely to change therapy [44]. Conversely, a NVP-based regimen may yield lower HIV viral load (<1 copy/mL) than an EFV-based regimen [45]. However, a systematic review recommended an EFV-based regimen as a first-line therapy over NVP for HIV patients in resource-limited settings owing to a lower risk of virological failure [43]. Nevertheless, in resource-limited settings a similar virological outcome has been reported for both EFV and NVP regimens despite the difference in their adverse events [46].

In general, data are still limited regarding changes in QoL related to ART regimens based on NVP or EFV. Previous studies from developed countries have studied QoL and ART regimens by comparing either NVP with protease inhibitor or EFV with a protease inhibitor. Among ART-experienced patients who were switched to either EFV or protease inhibitor, patients who received EFV reported a better QoL [37]. Another study with ART-experienced patients who either received NVP or a protease inhibitor reported no significant differences in immunological changes (CD4 count) between the two groups; however, the NVP group recorded a significantly better improvement in QoL than the protease inhibitor group [47]. One other study reported no differences in QoL between patients on NVP and EFV among HIV patients naïve to ART in a multisite study in Asia, South Africa, Australia, North America and Europe [4]. However, the majority of patients had a low CD4 count (<200 cells/μL) and their regimen included stavudine. The researchers also combined the results of the patients who received NVP once daily with those who received it twice a day [4].

 Adherence to ART has been reported in other studies to be associated with QoL [13, 48]. A modest or no association between adherence and QoL has also been reported [49, 50]. Interestingly, both groups in this study maintained high levels of adherence that were not significantly different despite that the fact that the EFV regimen is one combined pill once a day and the NVP regimen used in this study had to be taken twice a day. There was no significant association between QoL and adherence with the two ART regimens. High levels of adherence have been reported among Ugandans on ART (91–100 % adherence scores) [26, 51]. It is possible that adherence fatigue develops later on, since our study group had been on ART for only six months.

Among patients receiving a NVP-based ART regimen, depression score was the only variable associated with lower PHS and GPGI. Being female was associated with lower MHS. WHO stage 3&4, low education level and probable depression were associated with poor QoL in this study among patients on EFV. Depression and alcohol use had a significant negative association with global QoL among patients receiving an EFV-based regimen. Depression was independent of time during the follow-up period. Depression has been associated with poor QoL in PLHA in other studies in both resource-limited settings and developed countries [52–54]. In these studies, depression was associated with poor QoL, regardless of ART regimen. In this study, PLHA with a low level of education had poor QoL. Likewise, low education level has also been associated with poor QoL in other studies among patients receiving ART [19, 55, 56]. In this study, regardless of ART regimen, women reported lower QoL than men, and the same finding has also been reported in another studies [57, 58]. Higher WHO stage has also been reported to be a predictor of poor QoL among HIV patients in Ethiopia [58]. The interaction between WHO stage and time indicates that there are differences in recovery rates between the stages of HIV during the follow-up time.

### Limitations

 This was not a randomized study but future studies may build on these results. We recommend a randomized study with women with a CD4 count of <250 cells/μL and men with a CD4 count of <400/μL cells randomized to receive either NVP or EFV, with assessment of QoL as the main outcome measure. Follow-up of six months is short for a chronic illness and does not allow for assessment of regimen efficacy or the proportion of patients who fail to respond to therapy or switch to another regimen. We recommend studies with long follow-up and a large sample of patients initiating ART to confirm the continuous improvement in QoL that occur when ART is initiated at relatively high CD4 counts.

## Conclusions

There was a trend towards an increase in mean and median QoL scores among patients on ART in this study, especially with the EFV-based regimen. The changes in MHS and GPGI did not differ between patients using EFV or NVP as the first-line treatment from baseline to six months, and no difference was found in PHS for patients with a CD4 count >250. Patients with a very low CD4 count (<100 cells/μL) have better PHS while on a NVP regimen than an EFV regimen. By contrast, patients with a CD4 count between 101 and 250 cells/μL have a better PHS with an EFV regimen than a NVP regimen. Initiating an ART regimen containing EFV at a higher CD4 count led to improvement in QoL with time. NVP caused more severe toxicity and a relatively higher incidence of toxicities that warranted regimen change than EFV. This study supports the WHO guidelines that were in effect at the time of the study for resource-limited settings to initiate ART at a higher CD4 count and to use either a regimen based on NVP or EFV as a first-line therapy, albeit with a preference to EFV.

Regardless of ART regimen, women initiating ART may require more psychosocial support to improve their mental health. All PLHA initiating ART should be screened for depression and alcohol use, and appropriate support should be given in order to improve or maintain a high QoL.

## Abbreviations

3TC: Lamivudine
ART: Antiretroviral therapy
AZT: Zidovudine
CES-D: Center for Epidemiological Depression Scale
CI: Confidence interval
EFV: Efavirenz
GEE: Generalized estimating equations
GPGI: Global Person Generated Index
HRQoL: Health-related quality of life
MHS: Mental Health Summary score
MOS-HIV: Medical Outcomes Study-HIV
NVP: Nevirapine
PHS: Physical Health Summary Score
PLHA: People living with HIV/AIDS
QoL: Quality of life
SD: Standard deviation
TDF: Tenofovir
WHO: World Health Organization
